# Supplementary material for: A shark variable new antigen receptor recognizes an occluded epitope of fibroblast activation protein
Source: bioRxiv. 2026 Aug 12:2026.08.11.744296. Preprint. [Version 1] doi: 10.64898/2026.08.11.744296 (PMC13640736; doi:10.64898/2026.08.11.744296)
Supplement: Supplementary file 1 [file media-1.pdf]

A shark variable new antigen receptor recognizes an occluded epitope of fibroblast activation protein

Roma L. Broadberry[1,2], Kendahl L. Ott[3,4,5], Eric W. Lake[4,6], Tapanmitra Ravi[7], Colin A, Hemme[1,2], Shang Chi[1,2], Jayden L. West[4,5], Gihan S. Gunaratne[4], Irene M. Ong[7], Aaron M. LeBeau [4,6,7*], Timothy Grant [1,2*]

1. John and Jean Rowe Institute of Virology, Morgridge Institute of Research, Madison, WI 53715, USA

2. Department of Biochemistry, University of Wisconsin–Madison, Madison, WI 53715, USA

3. Department of Pathology and Laboratory Medicine, University of Wisconsin School of Medicine and Public Health, Madison, WI 53705, USA

4. Molecular and Cellular Pharmacology Program, University of Wisconsin School of Medicine and Public Health, Madison, WI, 53705, USA

5. Department of Radiology, University of Wisconsin School of Medicine and Public Health, Madison, WI 53705, USA

6. Department of Biostatistics and Medical Informatics, University of Wisconsin School of Medicine and Public Health, University of Wisconsin-Madison, Madison, WI, USA.

7. University of Wisconsin Carbone Cancer Center, University of Wisconsin School of Medicine and Public Health, Madison, WI 53705, USA

*Corresponding Authors:

Aaron M. LeBeau, University of Wisconsin, Madison, Wisconsin Institutes for Medical Research, 1111 Highland Avenue, Madison, WI 53705, USA. Email: aaron.lebeau@wisc.edu

Timothy Grant, University of Wisconsin, Madison, Morgridge Institute for Research, 330 N. Orchard St. Madison, WI 53715, USA. Email: tim.grant@wisc.edu

SUPPLEMENTAL FIGURES.

Supplemental Figure 1.

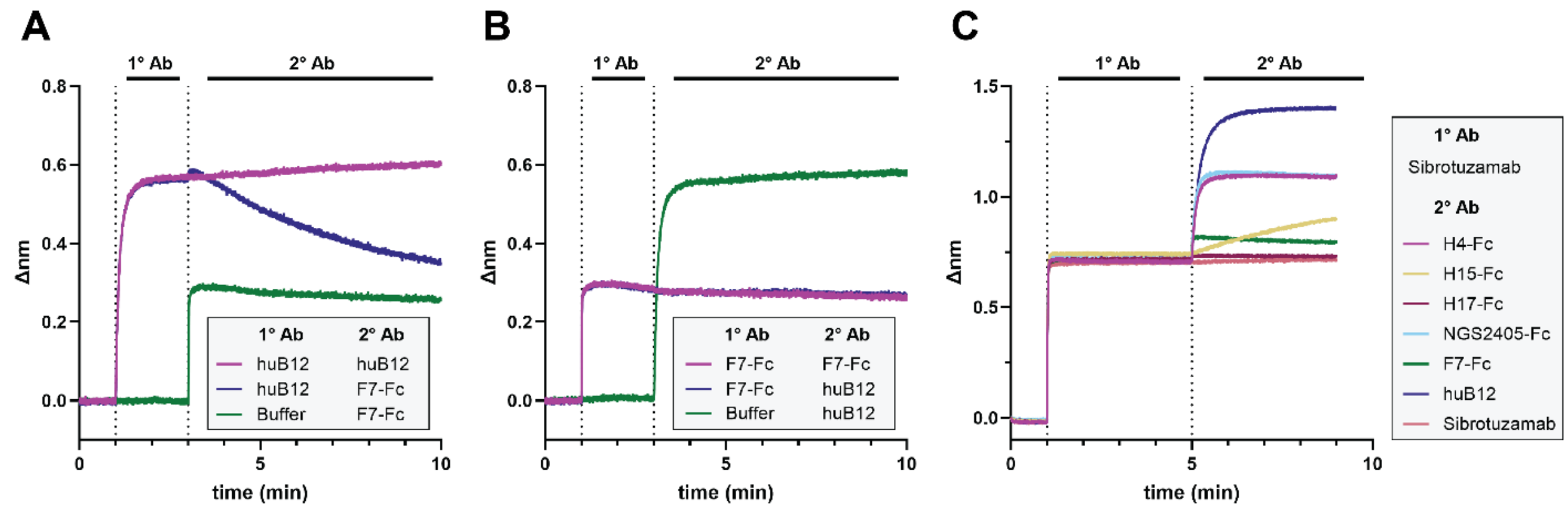

Supplemental Figure 1. Biolayer Interferometry (BLI) epitope binning of FAP antibodies. Binning of immobilized hFAP with (**A**) huB12, (**B**) F7-Fc, and (**C**) sibrotuzumab as the primary antibody, followed by the indicated competing antibodies.

Supplemental Figure 2.

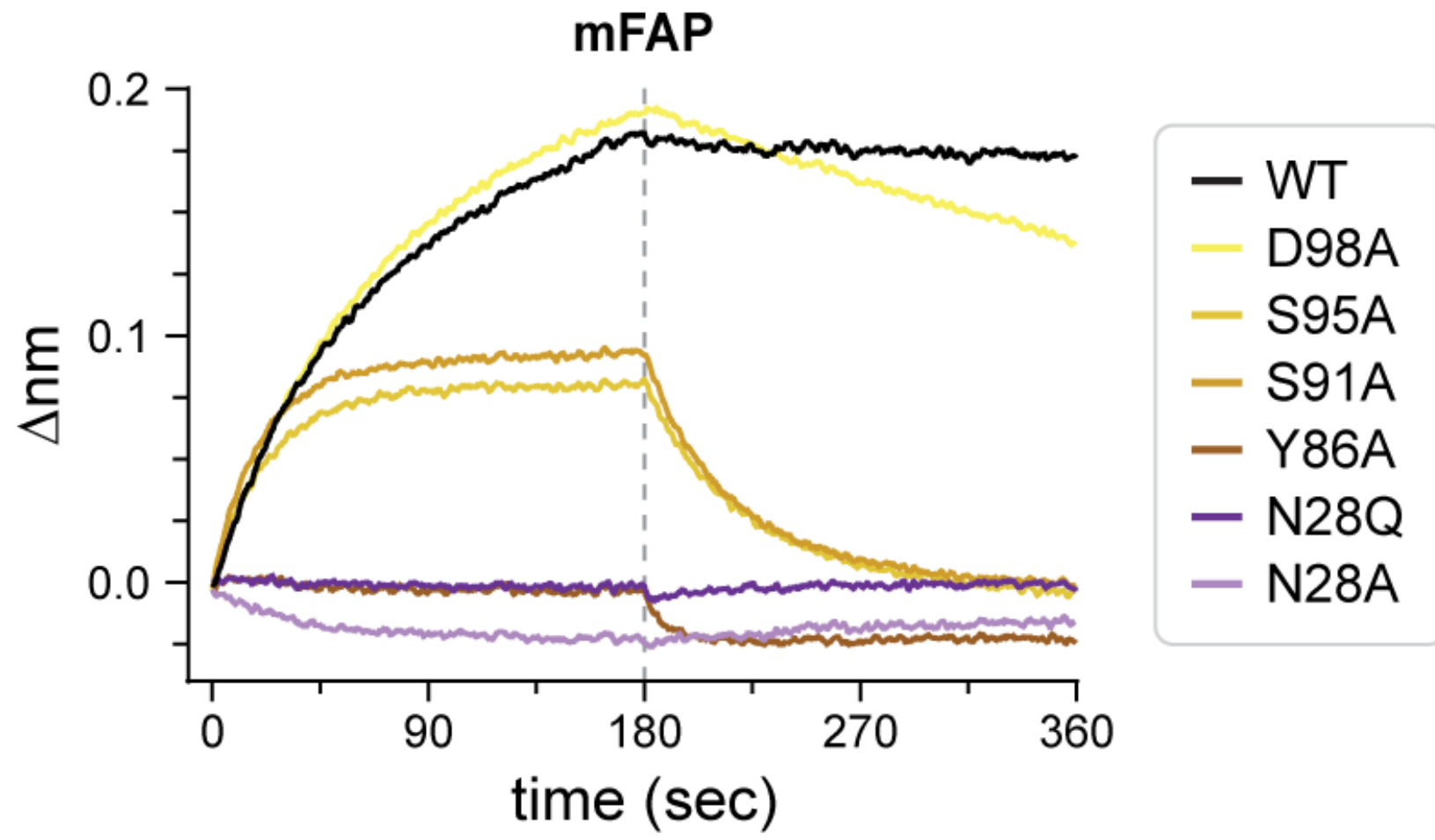

Supplemental Figure 2. BLI affinity readings of H4 binding to mFAP. Real-time association and dissociation sensorgrams for H4-Fc WT and point-mutant variants (250 nM) binding to mouse FAP (mFAP).

Supplemental Figure 3.

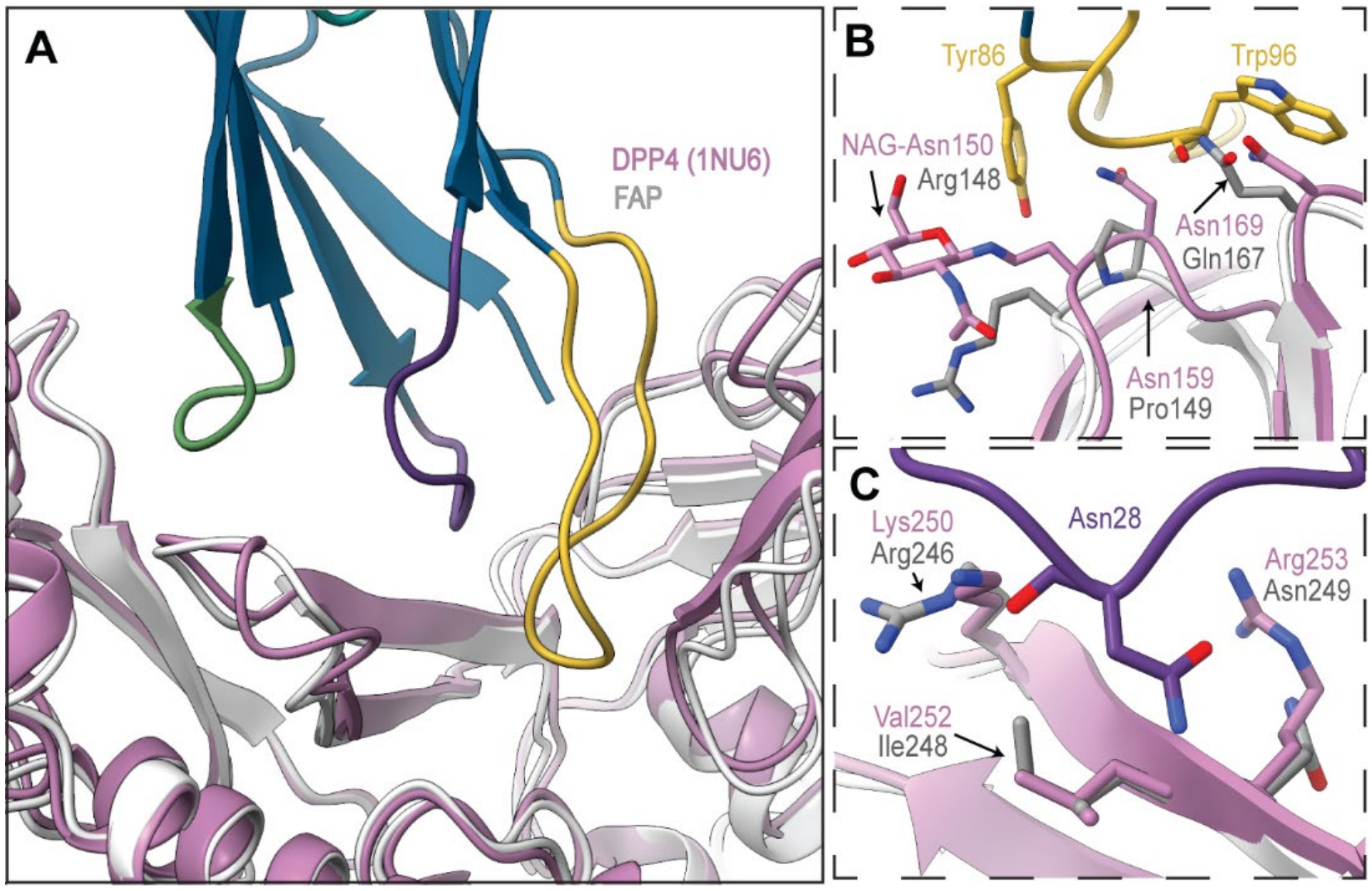

Supplemental Figure 3. DPP4 alignment with FAP-H4. Structural alignment of DPP4 (pink; PDB 1NU6)[1] onto the FAP–H4 complex (grayscale) (**A**) with H4 (blue) colored by CDR loop (CDR1, purple; CDR2, teal; CDR3, yellow). A close-up of the CDR3 interface (**B**) along with substitutions proximal to the CDR1-Asn28 interaction (**C**).

Supplemental Figure 4.

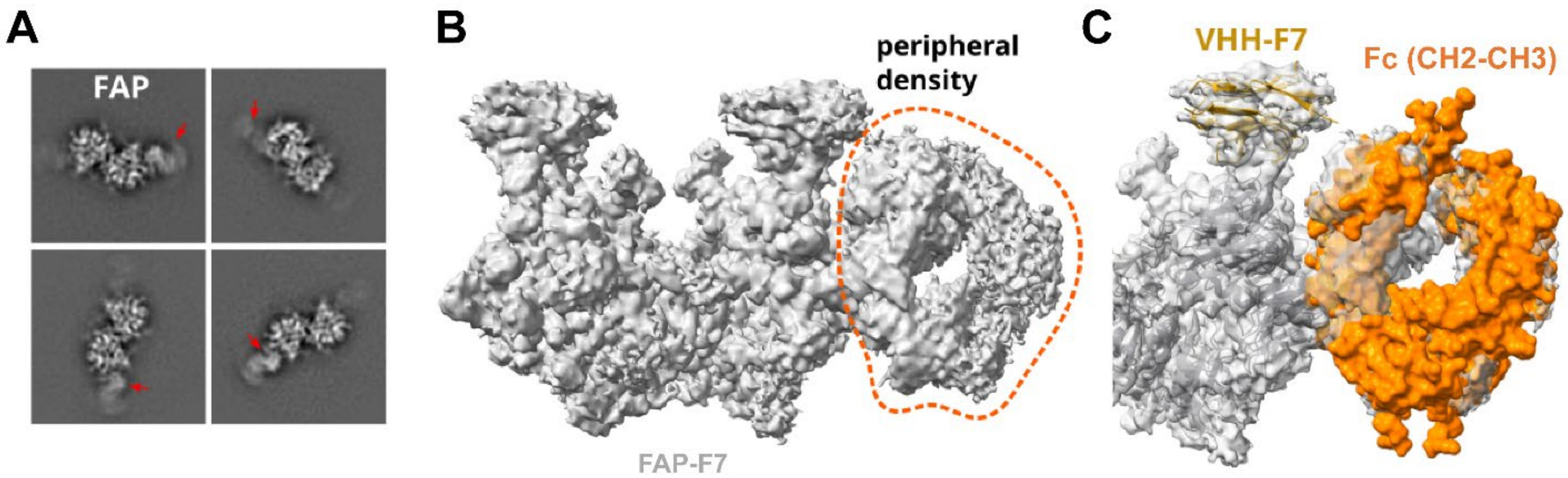

Supplemental Figure 4. Additional EM density observed in the FAP-F7 dataset. Two-dimensional classification of 1,520,217 picked particles identified a lower-resolution subset of 150,153 particles (9.9%) containing low-resolution density additional to the FAP-F7 data. (**A**) Representative 2D class averages showing this density adjacent to the FAP–F7 complex (red arrowheads). (**B**) Three-dimensional reconstruction of the corresponding particle subset, with the peripheral density outlined by the orange dashed contour; overall density is shown in gray. (**C**) The model fitting of fused Fc region predicted from Alpha-Fold[2] (orange) extending from the FAP-bound VHH-F7 domain (yellow).

Supplemental Fig. 5.

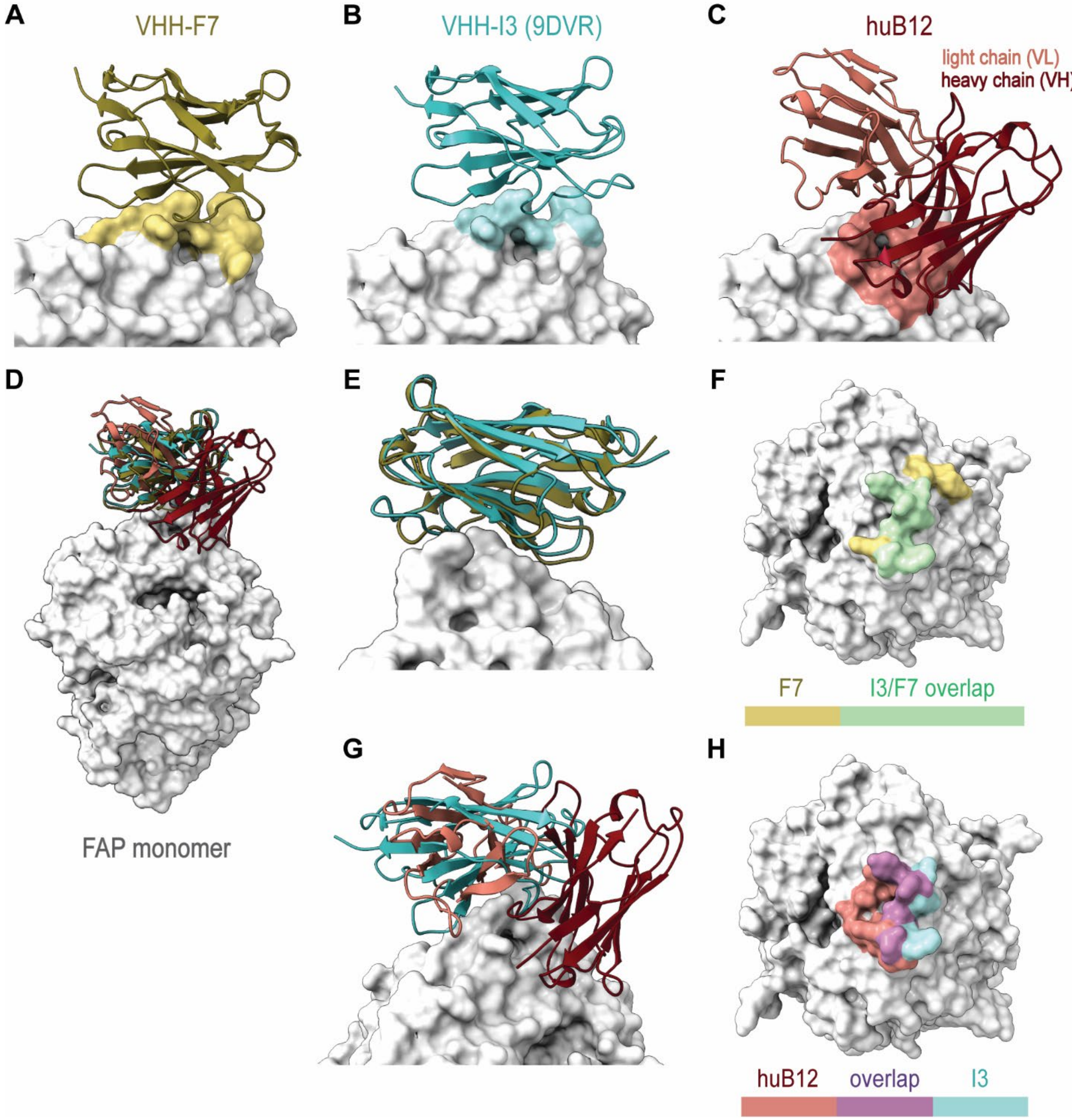

Supplemental Fig. 5. Structural alignment of FAP-bound F7, huB12, and I3-VHH. Binding modes for F7 (**A**), I3 (**B**, PDB: 9DVR),[3] and huB12 (**C**) mapped onto FAP monomer (grey), with corresponding epitope footprints colored on the FAP surface (yellow, cyan, red, respectively). Structural overlay of F7, I3, and huB12 on a single FAP monomer, showing convergence on the solvent-exposed β-propeller surface (**D**). Close-up alignment of F7 and I3 showing similar binding orientations (**E**) and the F7 epitope (yellow) completely overlaps the I3 epitope (green) (**F**). Overlay of F7, I3, and huB12 showing that huB12 engages the same general β-propeller region from a distinct orientation (**G**) with surface representation of the huB12 (red) and I3 (cyan) epitopes highlighting partial overlap (magenta) between the binding sites (**H**).

Supplemental Fig. 6.

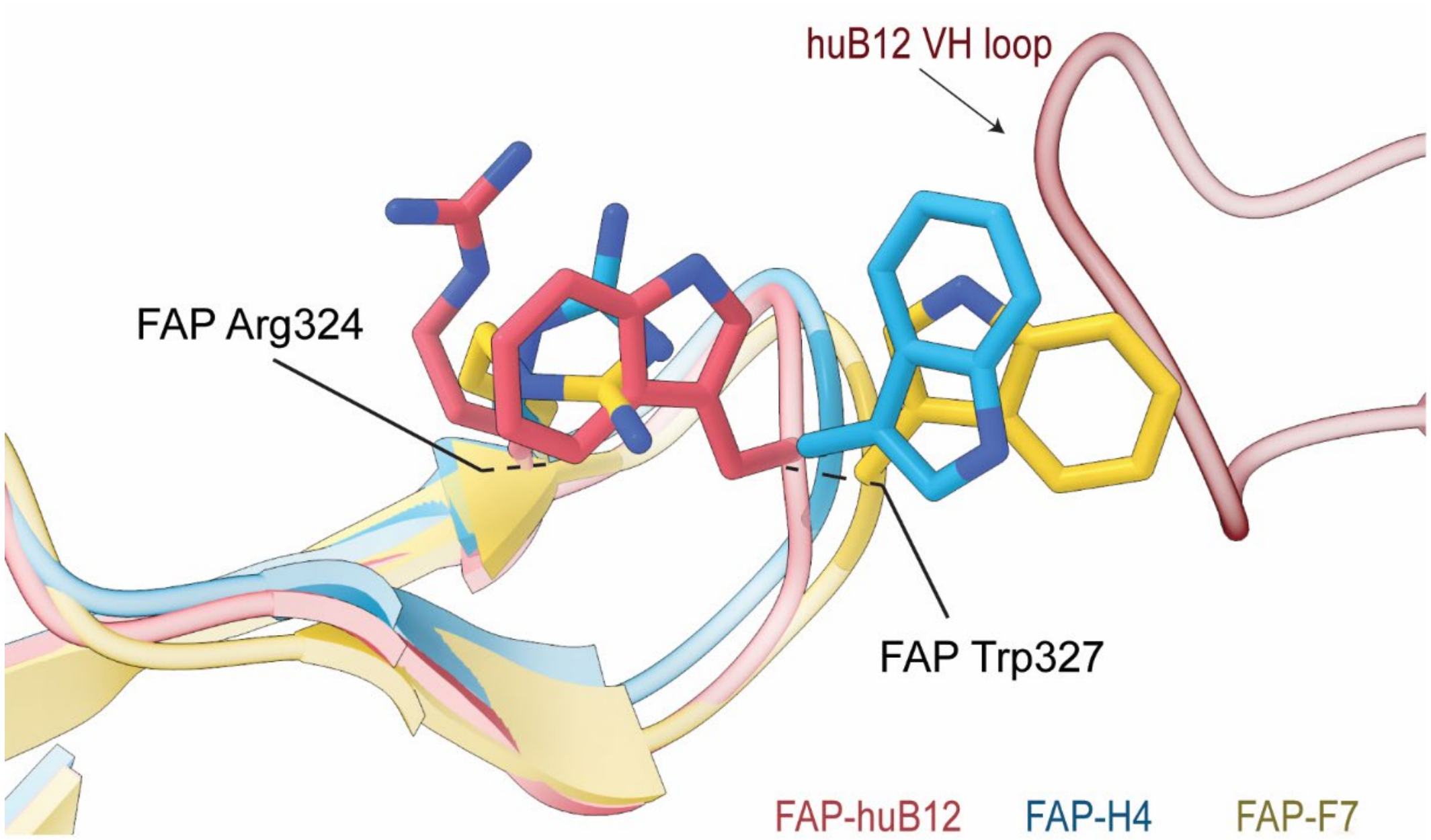

Supplemental Fig. 6. Local rearrangements of FAP Arg324 and Trp327. Superposition of the FAP-huB12, FAP-H4, and FAP-F7 structures showing the distinct conformations of FAP Arg324 and Trp327. FAP from the huB12, H4, and F7 complexes is shown in red, blue, and gold, respectively.

Supplemental Fig. 7.

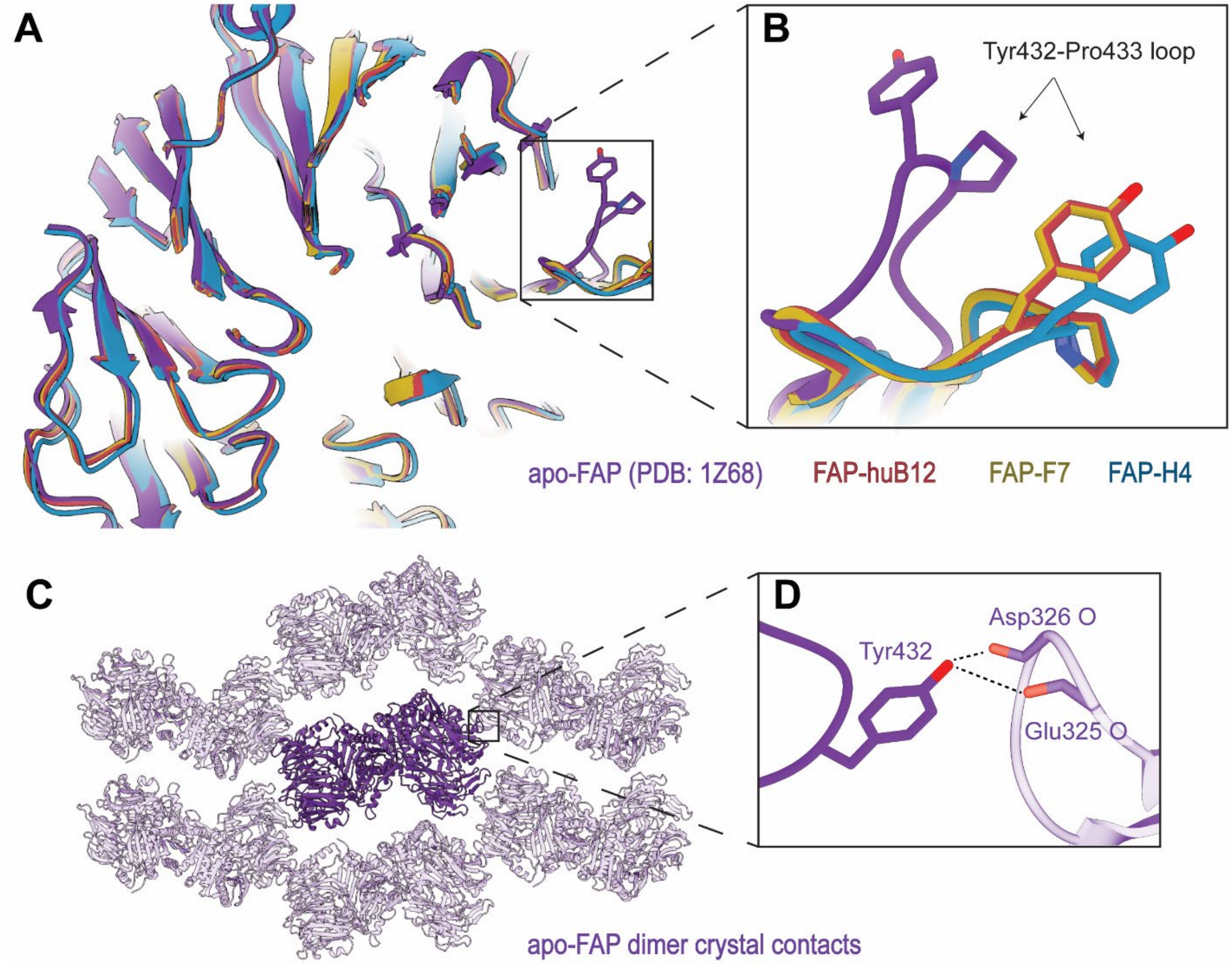

Supplemental Fig. 7. Crystal packing may stabilize an altered Tyr432–Pro433 loop conformation in apo-FAP. (**A**) Structural alignments of the outer β-propeller region from apo-FAP and the FAP-huB12, FAP-F7, and FAP-H4 structures. (**B**) Enlarged view of the Tyr432–Pro433 loop, highlighting its altered position in apo-FAP relative to the antibody-bound structures. Apo-FAP, FAP-huB12, FAP-F7, and FAP-H4 are shown in purple, red, gold, and blue, respectively. (**C**) Crystal-packing arrangement generated from the apo-FAP crystal structure (PDB 1Z68).[4] (**D**) Enlarged view of the crystal contact formed by Tyr432 of the focal FAP molecule and oxygen atoms (red) from Asp326 and Glu325 of a symmetry-related molecule. Dotted lines indicate intermolecular contacts within 3Å (ChimeraX).[5]

Supplemental Figure 8.

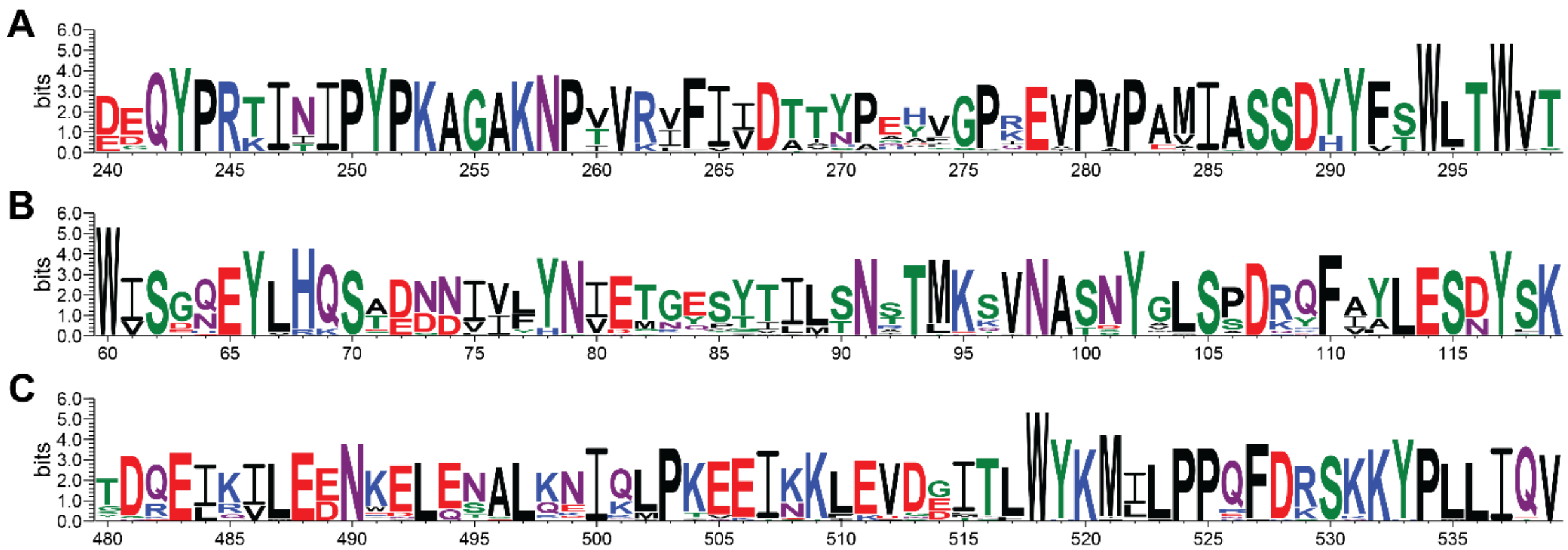

Supplemental Figure 8. Conservation of FAP sequences within antibody epitopes across mammalian species. Sequence logos generated from mammalian FAP amino acid sequences illustrate residue conservation within the mapped epitope regions of anti-FAP antibodies, **(A)** huB12, F7, and H4, **(B)** H15, and **(C)** NGS2405.

Supplemental Fig. 9.

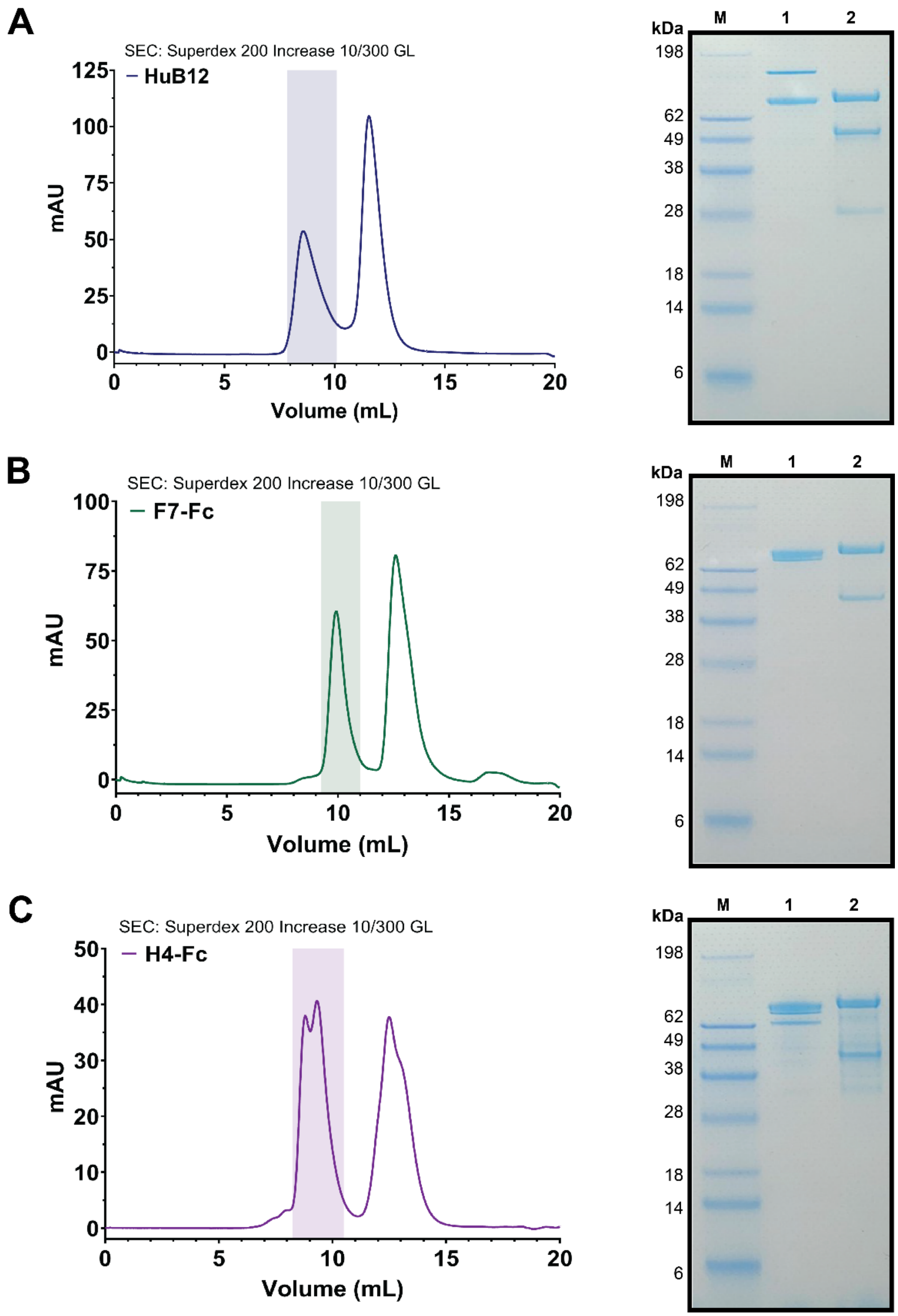

Supplemental Fig. 9. Size-exclusion chromatography and SDS-PAGE FAP complexes. Size-exclusion chromatography (left) of FAP in complex with huB12 (**A**), F7-Fc (**B**), or H4-Fc (**A**) with corresponding SDS-PAGE analyses of peak fractions (right) under non-reducing (1) and reducing conditions (2).

Supplemental Fig. 10.

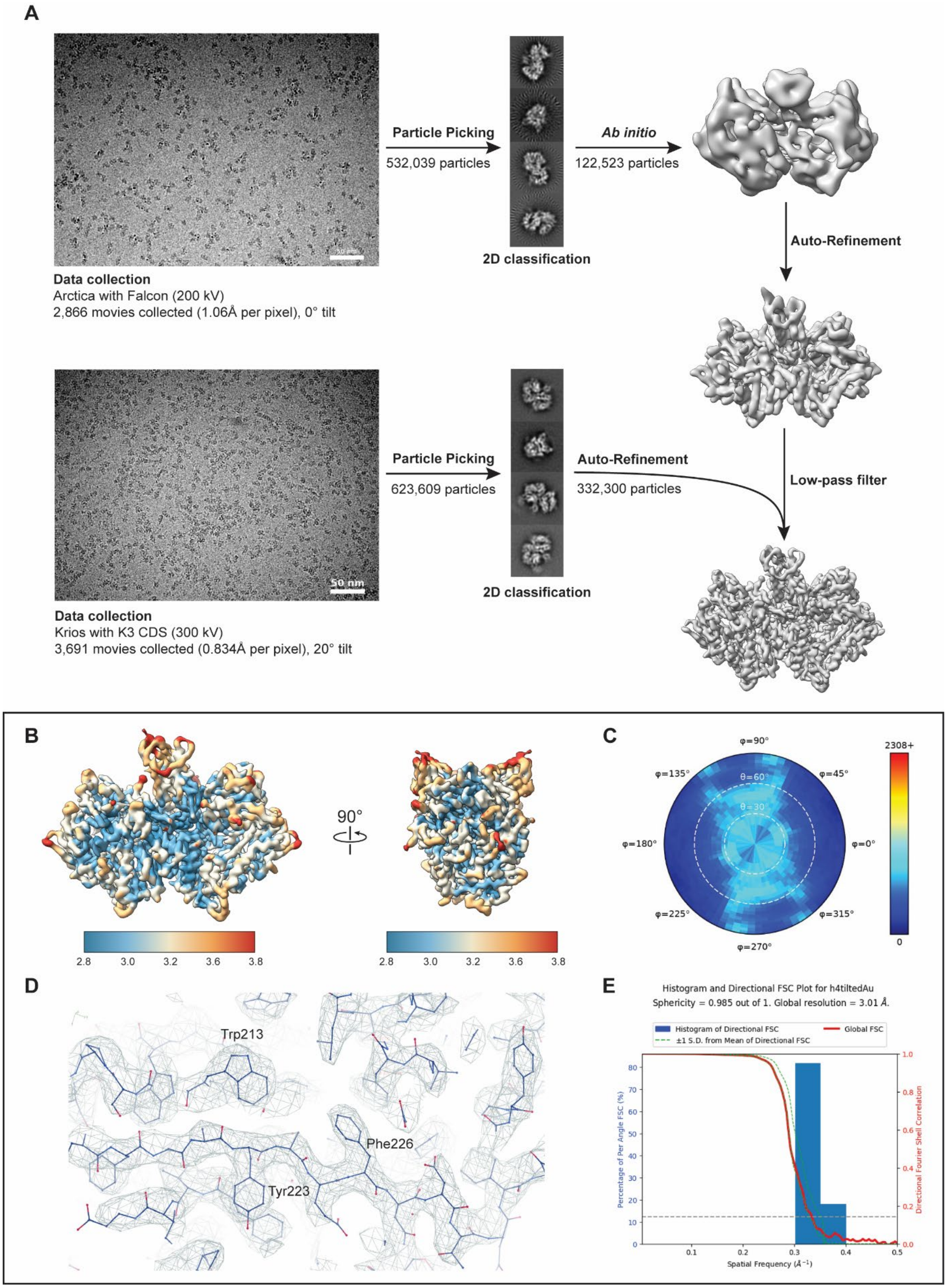

Supplemental Fig. 10. Cryo-EM processing workflow and validation of FAP-H4. (**A**) Representative micrographs, 2D class averages, and processing workflow for the FAP-H4 complex. (**B**) Local resolution estimates shown in two orthogonal views of the final reconstruction. (**C**) Angular distribution plot showing particle-view sampling in the final reconstruction. (**D**) Representative cryo-EM density with fitted atomic model, highlighting local map quality around selected aromatic residues. (**E**) All processing performed in *cisTEM*;[6] 3D Fourier shell correlation curve and directional FSC histogram for the final reconstruction.[7]

Supplemental Fig. 11.

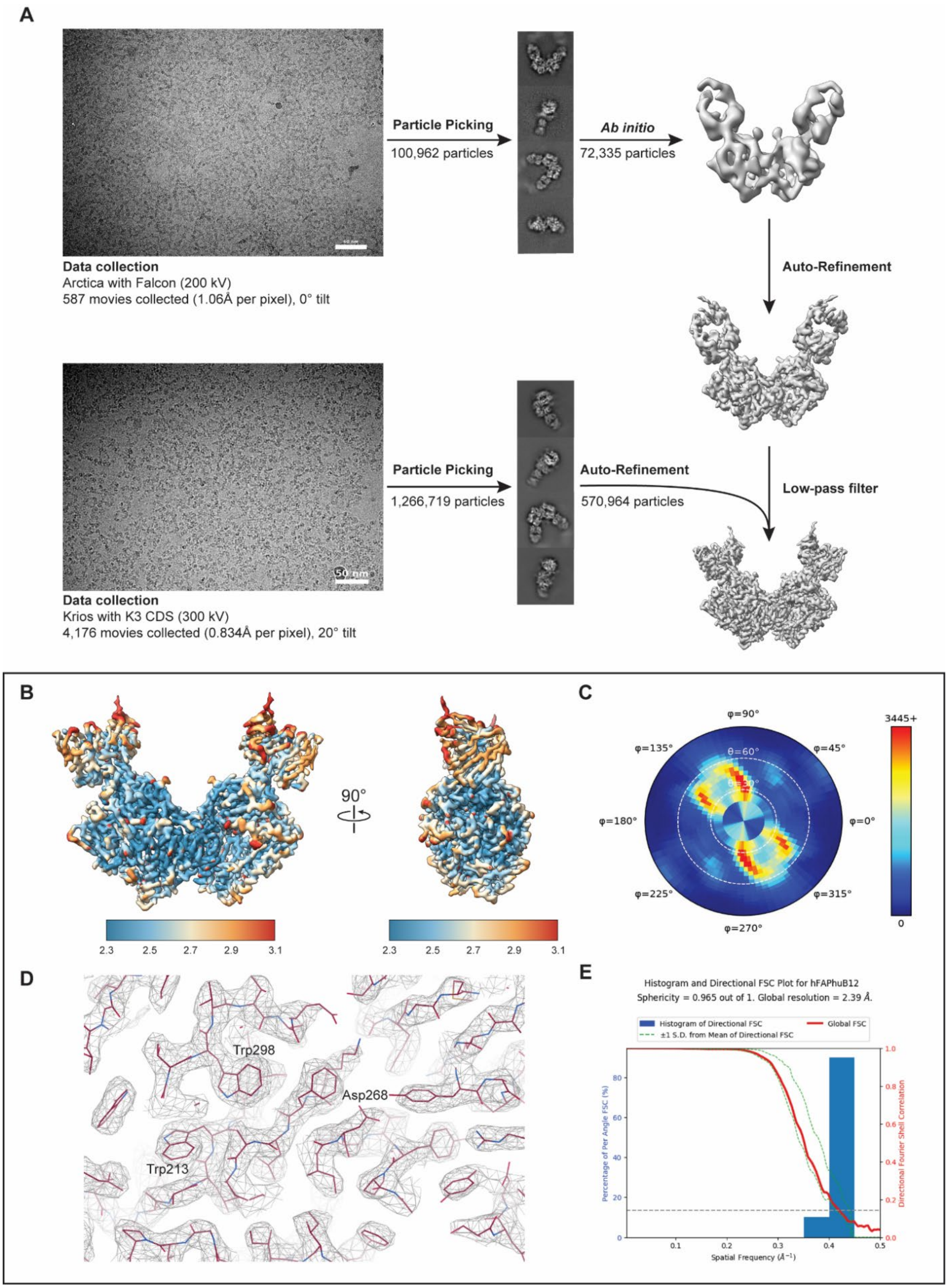

Supplemental Fig. 11. Cryo-EM processing workflow and validation of FAP-huB12. (**A**) Representative micrographs, 2D class averages, and processing workflow for the FAP-huB12 complex. (**B**) Local resolution estimates shown in two orthogonal views of the final reconstruction. (**C**) Angular distribution plot showing particle-view sampling in the final reconstruction. (**D**) Representative cryo-EM density with fitted atomic model, highlighting local map quality around selected aromatic residues. (**E**) All processing performed in *cisTEM*;[6] 3D Fourier shell correlation curve and directional FSC histogram for the final reconstruction.[7]

Supplemental Fig. 12.

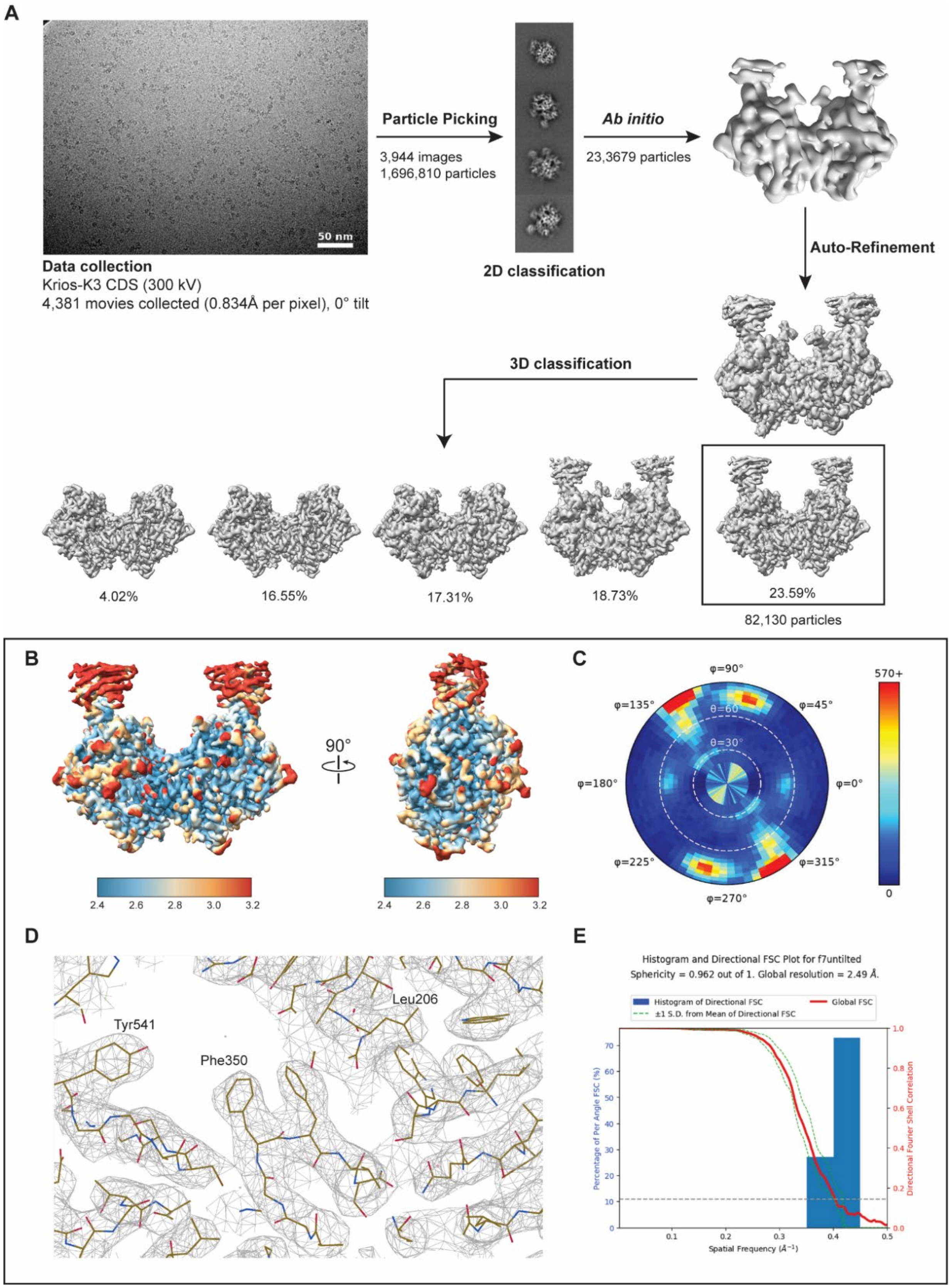

Supplemental Fig. 12. Cryo-EM processing workflow and validation of the FAP-F7-Fc reconstruction.
(**A**), Representative micrograph, 2D class averages, and processing workflow for the FAP-F7 complex. Particles were selected from 2D classification, used for ab initio model generation and auto-refinement, and further separated by 3D classification. The boxed 3D class was selected for final refinement. (**B**) Local resolution estimates for the final FAP-F7 reconstruction shown in two orthogonal views. (**C**), Angular distribution plot showing particle-view sampling. (**D**), Representative cryo-EM density with fitted atomic model, highlighting local map quality around selected residues. (**E**) All processing performed in *cisTEM*;[6] 3D Fourier shell correlation curve and directional FSC histogram for the final reconstruction.[7]

Table 1. Cryo-EM data acquisition and model statistics.

Statistic	FAP-H4 (EMD-78501/PDB: 37US)	FAP-huB12 (EMD-78502, PDB: 37UT)	FAP-F7 (EMD-78503, PDB: 37UU)
**Data collection and processing**			
Microscope	Krios-G3	Krios-G3	Krios-G3
Voltage (kV)	300	300	300
Detector	K3 (Counting)	K3 (Counting)	K3 (Counting)
Magnification (nominal/calibrated)	105,000	105,000	105,000
Data acquisition software	SerialEM	SerialEM	SerialEM
Electron exposure ($e^-/Å^2$)	80	80	80
Exposure rate (e^-/pixel/s)	7.7	7.7	7.7
Frames per micrograph	80	80	80
Pixel size (Å)	0.834	0.834	0.834
Defocus range (μm)	-0.5 to -2.0	-0.5 to -2.0	-0.5 to -2.0
Stage tilt (°)	20	20	0
Micrographs collected (no.)	3,691	4,176	4,381
**Reconstruction**			
Image-processing package	*cis*TEM	*cis*TEM	*cis*TEM
Total extracted particles (no.)	623,609	1,266,719	1,696,810
Final particles (no.)	332,300	570,964	82,130
Symmetry imposed	C2	C2	C2
Resolution (Å), FSC 0.143 (masked/unmasked)	2.9/3.0	2.4/2.6	2.5/2.8
3DFSC sphericity	0.985	0.965	0.962
**Model composition**			
Protein residues	1656	1882	1674
Nucleotides	0	0	0
Ligands / glycans	18 (NAG)	20 (NAG)	22 (NAG)
**Refinement**			
Refinement package	Phenix	Phenix	Phenix
CC (volume / mask)	0.79/0.83	0.85/0.85	0.8/0.81
Highest resolution used in refinement (Å)	3.5	3.5	3.5
**R.m.s. deviations**			
Bond lengths (Å)	0.003	0.005	0.005
Bond angles (°)	0.874	0.971	0.977
**Validation**			
Map-to-model FSC 0.5 (Å)	3.1	3.0	3.5
**Ramachandran plot**			
Outliers (%)	0	0	0
Allowed (%)	1.76	1.71	1.80
Favored (%)	98.24	98.29	98.20
MolProbity score	1.37	1.32	1.43
Poor rotamers (%)	0	0	0
Clashscore (all atoms)	6.67	5.85	7.92
C-beta deviations (%)	0	0	0
CaBLAM outliers (%)	1.22	2.05	2.05

Table 2. Sequence homology of human FAP (hFAP) across representative vertebrate species.

Species	hFAP Sequence Homology (%)
XP_013816149.1 *Apteryx mantelli*	74
XP_025950250.1 *Dromaius novaehollandiae*	74
XP_009674444.1 *Struthio camelus*	74
XP_009805927.1 *Gavia stellata*	74
XP_061860738.1 *Colius striatus*	74
XP_053926393.1 *Cuculus canorus*	73
XP_030310144.1 *Calypte anna*	74
XP_006037511.1 *Alligator sinensis*	73
XP_007421586.1 *Python bivittatus*	72
XP_020654035.2 *Pogona vitticeps*	72
XP_044274272.1 *Varanus komodoensis*	70
XP_031816435.1 *Sarcophilus harrisii*	87
XP_072468260.1 *Notamacropus eugenii*	85
NP_032012.1 *Mus musculus*	90
NP_620205.1 *Rattus norvegicus*	89
XP_040601389.1 *Mesocricetus auratus*	90
XP_016805441.1 *Pan troglodytes*	100
XP_004032755.2 *Gorilla gorilla gorilla*	100
NP_004451.2 *Homo Sapiens*	100
XP_003266222.1 *Nomascus leucogenys*	99
XP_003921979.1 *Saimiri boliviensis*	97
XP_073927060.1 *Castor canadensis*	94
NP_001091470.1 *Bos taurus*	94
XP_069430438.1 *Ovis canadensis*	94
XP_040085172.1 *Oryx dammah*	94
XP_019778827.2 *Tursiops truncatus*	94
XP_030708465.1 *Globicephala melas*	94
XP_007183240.3 *Balaenoptera acutorostrata*	93
XP_006196283.1 *Vicugna pacos*	94
XP_002924909.2 *Ailuropoda melanoleuca*	94
XP_027446978.1 *Zalophus californianus*	94
XP_005640309.2 *Canis lupus familiaris*	93
XP_011283649.1 *Felis catus*	95
XP_014587994.1 *Equus caballus*	94
XP_046515435.1 *Equus quagga*	94
XP_004375484.1 *Trichechus manatus latirostris*	95
XP_008256892.2 *Oryctolagus cuniculus*	95
XP_012615844.1 *Microcebus murinus*	95

XP_021121240.1 *Heterocephalus glaber*	93
XP_001512952.2 *Ornithorhynchus anatinus*	84
XP_069472422.1 *Ambystoma mexicanum*	65

Supplemental References

1. Thoma, R. *et al.* Structural Basis of Proline-Specific Exopeptidase Activity as Observed in Human Dipeptidyl Peptidase-IV. *Structure* **11**, 947–959 (2003).

2. Abramson, J. *et al.* Accurate structure prediction of biomolecular interactions with AlphaFold 3. *Nature* **630**, 493–500 (2024).

3. Xu, Z., Sinha, A., Pandya, D. N., Schnicker, N. J. & Wadas, T. J. Cryo-electron microscopy reveals a single domain antibody with a unique binding epitope on fibroblast activation protein alpha. *RSC Chem. Biol.* (2025) doi:10.1039/D4CB00267A.

4. Aertgeerts, K. *et al.* Structural and Kinetic Analysis of the Substrate Specificity of Human Fibroblast Activation Protein α. *Journal of Biological Chemistry* **280**, 19441–19444 (2005).

5. Pettersen, E. F. *et al.* UCSF ChimeraX: Structure visualization for researchers, educators, and developers. *Protein Science* **30**, 70–82 (2021).

6. Grant, T., Rohou, A. & Grigorieff, N. cisTEM, user-friendly software for single-particle image processing. *Elife* **7**, (2018).

7. Tan, Y. Z. *et al.* Addressing preferred specimen orientation in single-particle cryo-EM through tilting. *Nat. Methods* **14**, 793–796 (2017).
